# Crusted Scabies Mimicking Psoriasis in a Patient With Down Syndrome: A Case Report

**DOI:** 10.7759/cureus.111106

**Published:** 2026-06-18

**Authors:** Lamis El Yamani, Ouissal Hormi, Nassiba Zerrouki, Nada Zizi

**Affiliations:** 1 Dermatology, Mohammed VI University Hospital of Oujda, Oujda, MAR; 2 Dermatology, Allergology, and Venerology, Mohammed VI University Hospital of Oujda, Oujda, MAR

**Keywords:** crusted scabies, down syndrome, ivermectin, norwegian scabies, topical benzyl benzoate

## Abstract

Crusted scabies, also known as Norwegian scabies, is a rare but highly contagious variant of scabies characterized by extensive hyperkeratotic plaques and a heavy mite burden. It predominantly affects immunocompromised individuals or those with neurological or cognitive impairments, including individuals with Down syndrome.

We report the case of a 29-year-old man with Down syndrome who developed a progressive, treatment-resistant erythematosquamous eruption initially misdiagnosed as psoriasis. Topical corticosteroid therapy led to clinical worsening, with widespread hyperkeratotic and crusted plaques. Further evaluation revealed the presence of *Sarcoptes scabiei* mites and eggs on microscopic examination of skin scrapings. The patient was successfully treated with a combination of oral ivermectin, topical benzyl benzoate, and keratolytic agents, alongside environmental decontamination and the treatment of close contacts.

This case underscores the diagnostic challenge of crusted scabies in cognitively impaired patients and highlights the risk of misdiagnosis, particularly as psoriasis, which may delay appropriate treatment and increase the risk of outbreaks. Early recognition, microscopic confirmation, and aggressive multidrug therapy are essential for effective management.

## Introduction

Crusted scabies (Norwegian scabies) is a severe and highly contagious variant of scabies caused by *Sarcoptes scabiei* var. *hominis*. It is characterized by an extensive mite burden and hyperkeratotic skin lesions, typically occurring in immunocompromised individuals and patients with neurological or cognitive disorders [1,2]. Due to its atypical clinical presentation, crusted scabies can mimic several inflammatory dermatoses, particularly psoriasis, leading to diagnostic delays and inappropriate treatment [3].

Individuals with Down syndrome may be at an increased risk of developing crusted scabies because of immune dysregulation and cognitive impairment, which can contribute to the delayed recognition of symptoms and disease progression [4]. The clinical resemblance between crusted scabies and psoriasis represents a significant diagnostic challenge, especially when hyperkeratotic plaques predominate and pruritus is absent or overlooked [2,3].

We report a case of crusted scabies initially diagnosed as psoriasis in a patient with Down syndrome. This case highlights the importance of considering scabies in the differential diagnosis of refractory psoriasiform eruptions and emphasizes the need for early parasitological evaluation to prevent misdiagnosis and disease transmission.

## Case presentation

**Figure 1:**
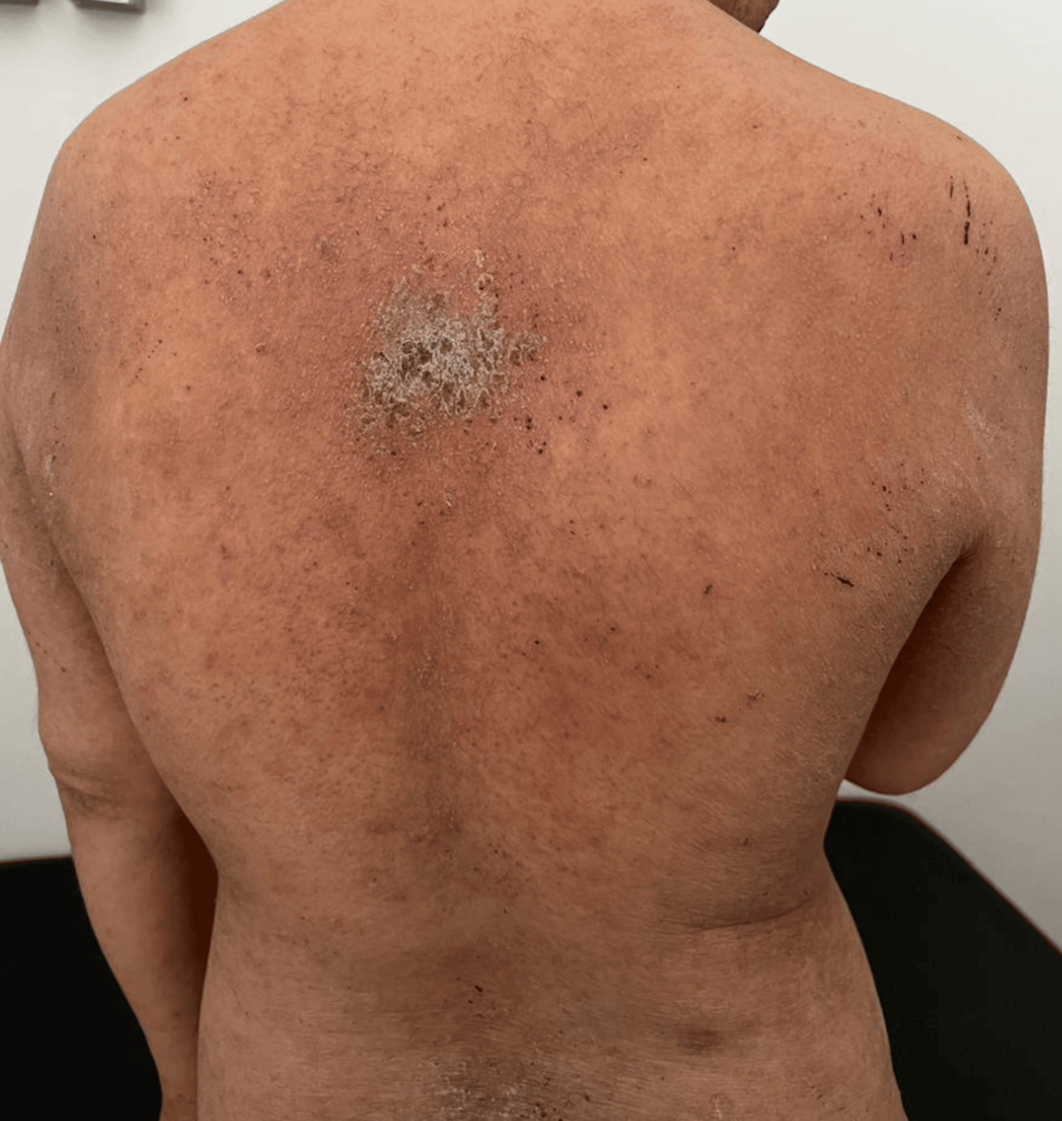
Thick hyperkeratotic plaque on the back in a patient with crusted scabies

**Figure 2:**
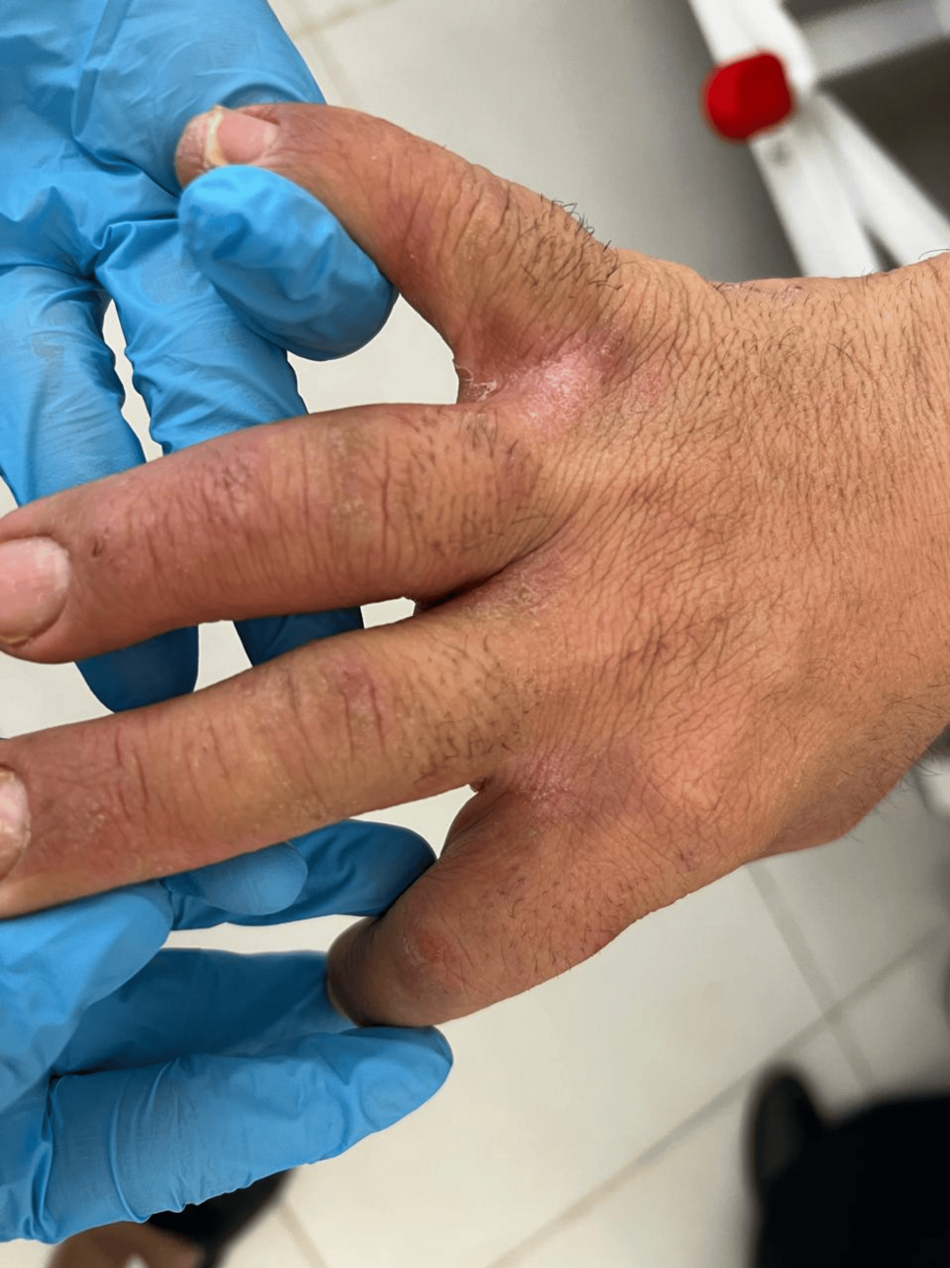
Interdigital erythema and scales in crusted scabies

A 29-year-old man with a history of Down syndrome with no other relevant history was referred to dermatology for the evaluation of a diffuse erythematosquamous eruption that had developed progressively over the past six months. He was treated with potent topical corticosteroids, keratolytic agents, and emollients under the assumption of psoriasis. However, despite compliance with the prescribed treatment, his skin condition significantly worsened, with increasing hyperkeratosis and the appearance of new lesions on previously unaffected areas. The plaques became more extensive and crusted, especially on the hands, feet, and extensor surfaces of the limbs. Given the lack of response and the progressive nature of the disease, he was referred to our dermatology department for further evaluation and management. At the time of referral, the patient's skin was diffusely involved with thick yellowish crusts, and his family members had also begun developing pruritic papules, raising concern for a possible contagious dermatosis. Clinical examination revealed thick, yellowish, and hyperkeratotic plaques, particularly on the elbows and knees, as well as on the trunk, scalp, and upper and lower limbs, with relative sparing of the face. The interdigital spaces of the hands showed pronounced erythematous lesions (Figures 1, 2).

Diffuse xanthonychia and subungual hyperkeratosis affected both fingernails and toenails. Pruritus was mild and inconsistently reported due to the patient's limited communication abilities. A diagnosis of crusted scabies was suspected based on a combination of clinical findings, including the widespread distribution of hyperkeratotic plaques, the lack of therapeutic response to topical corticosteroids, and the onset of pruritic lesions among family members. Skin scrapings were collected from affected areas and examined under light microscopy. Potassium hydroxide (KOH) preparations revealed a massive infestation, with numerous mites, eggs, and fecal pellets clearly visible. In parallel, the patient underwent an immunodeficiency workup to investigate possible predisposing factors. This included a complete blood count (CBC), serum immunoglobulin levels, and HIV serology, all of which were within normal limits or negative.

Several differential diagnoses were considered, including psoriasis, eczema, seborrheic dermatitis, and other hyperkeratotic dermatoses. Psoriasis was initially suspected because of the erythematous and scaly plaques involving the extensor surfaces. However, the progressive worsening despite potent topical corticosteroid therapy, the extensive crusted hyperkeratosis, the involvement of the interdigital spaces and nails, and the development of pruritic lesions in close family contacts were not consistent with psoriasis and favored an infectious parasitic dermatosis. Eczema and seborrheic dermatitis were considered less likely given the diffuse distribution and marked hyperkeratotic nature of the eruption. The diagnosis of crusted scabies was confirmed by direct microscopic examination.

**Figure 3:**
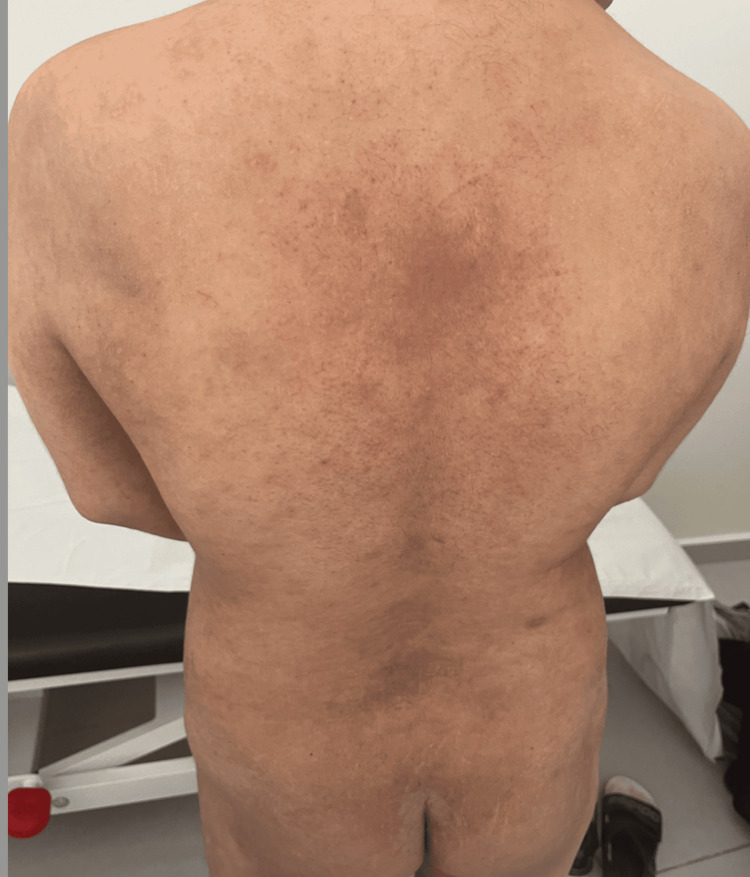
Complete resolution of the hyperkeratotic plaques after the treatment of crusted scabies

The patient was treated with oral ivermectin at a dose of 200 μg/kg on days 1, 2, and 8, in combination with topical benzyl benzoate 10% applied for seven consecutive days. A keratolytic treatment consisting of 30% salicylic acid in petroleum jelly was applied to the thickest crusted lesions to enhance the penetration of the topical scabicide. In addition, the decontamination of the environment was undertaken, including washing bedding and clothing at high temperatures. All close contacts received treatment, and the patient was placed under isolation during the contagious period to prevent transmission. Within three weeks, significant clinical improvement was noted, with the resolution of hyperkeratosis and pruritus (Figure 3). No secondary cases were reported among close contacts.

## Discussion

Crusted scabies represents the most severe form of infestation by *Sarcoptes scabiei* and is characterized by an exceptionally high parasite burden, often reaching millions of mites, compared with the few dozen mites usually found in classical scabies [5]. Although uncommon, it constitutes a major public health concern because of its extreme contagiousness and its potential to cause institutional and familial outbreaks when diagnosis is delayed [6].

The present case illustrates the diagnostic challenge posed by crusted scabies when it presents with extensive hyperkeratotic and erythematosquamous plaques resembling psoriasis. Psoriasiform presentations are among the most frequently reported clinical mimickers of crusted scabies, particularly in patients with limited communication abilities or atypical symptoms [7]. In our patient, the predominance of thick crusted plaques involving the extensor surfaces, scalp, trunk, and nails initially favored a diagnosis of psoriasis. Furthermore, the absence of intense pruritus, considered a hallmark of conventional scabies, contributed to the diagnostic delay. Similar observations have been reported in previous studies, emphasizing that pruritus may be absent, minimal, or underreported in crusted scabies, especially in neurologically impaired patients [8].

Individuals with Down syndrome appear to be particularly vulnerable to severe forms of scabies. Several mechanisms have been proposed, including impaired cellular immunity, altered T-cell function, and cognitive deficits that may delay symptom recognition and reporting [9]. Numerous case reports have documented crusted scabies in patients with Down syndrome, suggesting that this population should be considered at increased risk when presenting with diffuse hyperkeratotic dermatoses [10]. In our patient, no acquired immunodeficiency was identified, supporting the hypothesis that Down syndrome itself may represent a predisposing factor.

Nail involvement, as observed in our case with diffuse xanthonychia and subungual hyperkeratosis, is a well-recognized but often overlooked manifestation of crusted scabies. Mites may colonize the subungual space, creating a reservoir that contributes to persistence and recurrence if not adequately treated [11]. The coexistence of nail changes and extensive hyperkeratotic lesions should therefore raise suspicion for crusted scabies, particularly when conventional treatments for psoriasis fail.

The diagnosis of crusted scabies relies on the demonstration of mites, eggs, or scybala. In contrast to classical scabies, parasitological confirmation is generally straightforward because of the massive mite burden [2]. In our patient, the microscopic examination of skin scrapings rapidly established the diagnosis and avoided further inappropriate treatment. This observation highlights the importance of performing simple parasitological investigations in any refractory psoriasiform eruption, especially when epidemiological clues such as affected family members are present.

Crusted scabies represents a diagnostic challenge because its clinical presentation can closely resemble other inflammatory and hyperkeratotic dermatoses, particularly psoriasis [2,3,5,7]. Misdiagnosis is common, especially when the characteristic features of classical scabies are absent or overlooked [2,3]. In our patient, the initial diagnosis of psoriasis led to treatment with topical corticosteroids without clinical improvement. The marked hyperkeratosis, extensive crusting, nail involvement, and subsequent occurrence of pruritic lesions among household contacts ultimately raised suspicion of crusted scabies. This case highlights the importance of considering crusted scabies in the differential diagnosis of diffuse hyperkeratotic eruptions, particularly in individuals with Down syndrome, who may be predisposed to severe infestations and in whom delayed diagnosis can facilitate disease progression and transmission [5,9,10].

Current management guidelines recommend combining systemic and topical therapies, as topical treatment alone is often insufficient to eradicate the high mite burden [12]. For crusted scabies, the standard regimen combines a topical scabicide applied daily for seven days and then twice weekly until cured, with oral ivermectin at 200 μg/kg administered on days 1, 2, and 8. In severe cases, additional ivermectin doses are recommended on days 9 and 15 and may be extended to days 22 and 29 in the most severe or refractory cases. Keratolytic agents may be used to remove thick crusts and improve the penetration and efficacy of topical scabicides [12,13]. The favorable outcome observed in our patient following ivermectin, benzyl benzoate, and salicylic acid therapy is consistent with previous reports. Equally important are environmental decontamination measures and the simultaneous treatment of close contacts, which are essential to interrupt transmission and prevent reinfestation [6,12,13].

This case underscores the importance of considering crusted scabies in the differential diagnosis of generalized hyperkeratotic dermatoses, particularly in patients with Down syndrome or other conditions associated with cognitive impairment. Early recognition and parasitological confirmation are crucial to avoid prolonged morbidity, inappropriate immunosuppressive treatments, and the risk of outbreaks among close contacts and healthcare workers.

## Conclusions

Crusted scabies is an uncommon but severe manifestation of *Sarcoptes scabiei* infestation that may closely resemble psoriasis and other hyperkeratotic dermatoses, leading to delayed diagnosis and inappropriate treatment. Patients with Down syndrome appear to be particularly vulnerable because of underlying immune dysfunction and cognitive impairment, which may contribute to the delayed recognition of symptoms and disease progression. Our case emphasizes the importance of considering crusted scabies in the differential diagnosis of refractory psoriasiform eruptions, especially when lesions worsen despite topical corticosteroid therapy and when epidemiological clues, such as affected family members, are present. Early parasitological confirmation remains essential for establishing the diagnosis and initiating appropriate therapy. Prompt treatment combining systemic and topical scabicides, together with environmental decontamination and the management of close contacts, can result in favorable outcomes while preventing further transmission.
